# Supplementary material for: Resolving the full spectrum of human genome variation using Linked-Reads
Source: Genome Res. 2019 Apr;29(4):635–45. doi: 10.1101/gr.234443.118 (PMC6442396; doi:10.1101/gr.234443.118)
Supplement: Supplemental Material [file supp_gr.234443.118_Supplemental_longranger-2.2.zip › longranger-2.2.tar.bz2!/lr2.2-public/lib/stan/cmdstan/stan/doc/api/html/logical__eq_8hpp_source.html]

Stan: src/stan/math/functions/logical\_eq.hpp Source File


|  |  |
| --- | --- |
|  | Stan  2.5.0  probability, sampling & optimization |


- Main Page
- Related Pages
- Namespaces
- Classes
- Files

- File List
- File Members

All Classes Namespaces Files Functions Variables Typedefs Enumerations Enumerator Friends Macros Pages

- src
- stan
- math
- functions

logical\_eq.hpp

Go to the documentation of this file.

1 #ifndef STAN\_\_MATH\_\_FUNCTIONS\_\_LOGICAL\_EQ\_HPP

2 #define STAN\_\_MATH\_\_FUNCTIONS\_\_LOGICAL\_EQ\_HPP

3

4 namespace stan {

5  namespace math {

6

17  template <typename T1, typename T2>

18  inline int

19  logical\_eq(const T1 x1, const T2 x2) {

20  return x1 == x2;

21  }

22

23  }

24 }

25

26 #endif

stan::math::logical\_eq

int logical\_eq(const T1 x1, const T2 x2)

Return 1 if the first argument is equal to the second.

**Definition:** logical\_eq.hpp:19


---

[ Stan Home Page ]

*© 2011–2014,
Stan Development Team.*
